# Gender inequalities in heat-related mortality in the Czech Republic

**DOI:** 10.1007/s00484-023-02507-2

**Published:** 2023-07-10

**Authors:** Chloé Vésier, Aleš Urban

**Affiliations:** 1 Faculty of Environmental Sciences, Czech University of Life Sciences, Kamycka 129, 165 00 Prague, Czech Republic; 2 Institute of Atmospheric Physics of the Czech Academy of Sciences, Boční II 1401, 141 00 Prague, Czech Republic

**Keywords:** Mortality, Heat stress, Sex and gender inequalities, DLNM, Czech Republic

## Abstract

It is acknowledged that climate change exacerbates social inequalities, and women have been reported as more vulnerable to heat than men in many studies in Europe, including the Czech Republic. This study aimed at investigating the associations between daily temperature and mortality in the Czech Republic in the light of a sex and gender perspective, taking into account other factors such as age and marital status. Daily mean temperature and individual mortality data recorded during the five warmest months of the year (from May to September) over the period 1995–2019 were used to fit a quasi-Poisson regression model, which included a distributed lag non-linear model (DLNM) to account for the delayed and non-linear effects of temperature on mortality. The heat-related mortality risks obtained in each population group were expressed in terms of risk at the 99th percentile of summer temperature relative to the minimum mortality temperature. Women were found generally more at risk to die because of heat than men, and the difference was larger among people over 85 years old. Risks among married people were lower than risks among single, divorced, and widowed people, while risks in divorced women were significantly higher than in divorced men. This is a novel finding which highlights the potential role of gender inequalities in heat-related mortality. Our study underlines the relevance of including a sex and gender dimension in the analysis of the impacts of heat on the population and advocates the development of gender-based adaptation policies to extreme heat.

**Supplementary Information:**

The online version contains supplementary material available at 10.1007/s00484-023-02507-2.

## Introduction

With climate change, temperatures in Central Europe are expected to rise (IPCC 2021) and heatwaves to be more frequent and intense (Lhotka et al. 2018; IPCC 2021) during the twenty-first century. The latest data from the Met Office Hadley Centre (CCAG 2022) suggested that recent record-breaking European summers of 2018 (Hoy et al. 2020), 2019 (Xu et al. 2020) or 2021 (Lhotka and Kyselý 2022) may become the norm by 2035.

Exposure to high temperatures may affect human health, as it was shown to cause various heat-related illnesses (Székely et al. 2015; Watts et al. 2019) and may ultimately lead to premature death. Increase of mortality during heatwaves was already observed in many studies (Jonsson and Lundgren 2015; Xu et al. 2016), and heat-related mortality was predicted to increase by the end of the century, in particular in Central Europe (Gasparrini et al. 2017). In the Czech Republic, Urban et al. (2022) showed that, despite a decrease in the impacts of heat on mortality in the 1990s and 2000s compared to the 1980s (Kyselý and Kříž 2008; Kyselý and Plavcová 2012), the heat-related mortality risk was twice as high in the 2010s than in the previous three decades in Prague.

Not all humans are impacted the same way by short- and long-term increase in temperatures. In particular, inequalities in heat-related mortality based on sex, describing the biological and physiological characteristics of an individual, and gender, referring to norms, behaviours, values, and preferences considered appropriate by a society for each sex (Charkoudian and Stachenfeld 2014; Tenglerová et al. 2020; WHO 2021; UN High Commissioner for Refugees 2021), have been observed in many studies. Indeed, women were found at higher risk to die as a result of high temperatures in various European countries, such as in France (Canouï-Poitrine et al. 2006), Italy (Stafoggia et al. 2006; Ellena et al. 2020a), Germany (Gabriel and Endlicher 2011), Spain (Achebak et al. 2018; Marí-Dell’Olmo et al. 2019) and Scotland (Wan et al. 2022). In the Czech Republic, a higher mortality increase was observed in women than in men during the hot summer periods of 1982–2000 (Kyselý 2004), as well as during the four heatwaves of 2003 (Kyselý and Kříž 2008). Hůnová et al. (2017) studied the effects of the summer heatwaves of August 2003 and July 2006 on health in Prague and found a higher mortality risk for women than for men. Several studies were conducted on the effects of hot spells on cardiovascular mortality in the Czech Republic (Kyselý et al. 2011; Davídkovová et al. 2014; Hanzlíková et al. 2015) or regions of the country (Urban et al. 2016) and also found higher excess mortality in females than in males.

Yet, individuals cannot be characterised by their sex and gender only, and a multitude of other criteria, such as age or socioeconomic factors, could be taken into account. Advanced age was identified as an important driver of heat vulnerability (Hajat et al. 2007; Yu et al. 2010; Kenny et al. 2010; Arbuthnott and Hajat 2017; Watts et al. 2019; Son et al. 2019; Watts et al. 2021; de Schrijver et al. 2022; Wan et al. 2022), and elderly women were repeatedly found to be particularly vulnerable to high temperatures (Canouï-Poitrine et al. 2006; Hajat et al. 2007; Yu et al. 2010; Ellena et al. 2020a; Wan et al. 2022). As for socioeconomic status, factors such as the level of education (Hondula et al. 2012; Aubrecht and Özceylan 2013; Klein Rosenthal et al. 2014; Huang et al. 2015; Ellena et al. 2020a, 2020b), the marital status (Fouillet et al. 2006; Gronlund et al. 2015; Ellena et al. 2020a, 2020b; Wan et al. 2022), or the household structure (Seebaß 2017, Ellena et al. 2020a, 2020b) were sometimes found to be modifiers of heat effects.

Understanding differences in population exposure and vulnerability to heat conditions seems to be essential to develop relevant adaptation policies (Putnam et al. 2018). Considering the existing sex and gender inequalities in the society, integrating a sex and gender dimension into the analysis and the discussion is necessary to avoid concealing and to understand the differential impacts of heat on men and women. To date, no study investigated heat-related mortality in the Czech Republic with a primary focus on sex and gender, including combinations of sex with other criteria, such as marital status, as potential drivers of heat vulnerability.

The objective of this study was to quantify heat-related mortality and to identify potential sex and gender inequalities in heat vulnerability in the Czech Republic over the period 1995–2019 (25 years). Hence, differentiated analyses were conducted for men and women, and these categories were also studied while interlinked with the age category and the marital status. Ultimately, the fraction and number of deaths attributable to heat were evaluated in order to account for the actual burden of heat in the total mortality of each population group.

## Methods

### Study design and data

The study period extended from January 1, 1995, to December 31, 2019, for a total of 25 years, although only the five warmest months of the year (from May 1 to September 30, hereafter called “summer months”) were used in the analysis to focus on the highest temperatures. The study area was the Czech Republic, a 79,000 km^2^-wide country located in Central Europe, whose population size was relatively constant over the study period (10.3 million inhabitants in 1995 to 10.7 million in 2019) ([dataset] CZSO, Czech Statistical Office 2021). The analysis was chosen to be performed at the scale of the country rather than region- or city-wide, as it was shown that considering areas less populated than Prague in the Czech Republic would not give a robust and significant assessment of the heat-related mortality (Urban et al. 2016). Moreover, as pointed out by Urban et al. (2020), the Czech population is comparable to those of the largest European urban areas (e.g. Paris, London) and most of the Czech population lives within the same climate zone; hence, a nation-wide analysis would provide results comparable to studies from largest European cities.

In order to study the associations between heat and mortality, two types of time series were used: daily mortality and daily average temperature in the country.

The records of individual deaths were provided by the Institute of Health Information and Statistics of the Czech Republic (UZIS) and the Czech Statistical Office (CZSO). A total of 1,079,302 deaths were recorded during the summer months of the study period and were aggregated into daily mortality data. The data was divided by sex as a binary variable: “F” (female) and “M” (male). These two categories were further divided by age and by marital status, according to the following sub-categories: “0–64”, “65–74”, “75–84”, and “85+” for age, and “MAR” (married), “DIV” (divorced), “SIN” (single), and “WID” (widowed) for marital status. No distinction was made between heterosexual marriage and registered partnership in the data (same-sex couples are only entitled to registered partnership since 2006). As the number of deceased involved in registered partnerships (whether married, divorced, or widowed) accounted for less than 0.01% of the data, the results on marital status were analysed and discussed within the scope of heterosexuality only. Marital status was used as an indicator of the individual socioeconomic status; other indicators were not available in the original dataset, because they were either not recorded (i.e. household structure, income) or corrupted (i.e. level of education).

**Fig. 1:**
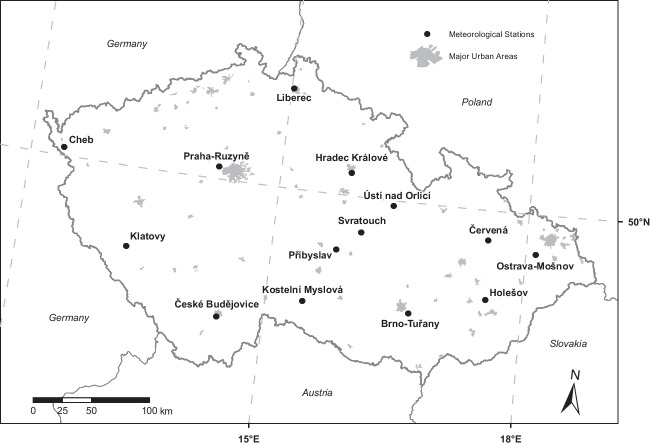
Map of the Czech Republic indicating the location of the 14 meteorological stations that provided the daily mean temperature used in this study and the major urban areas

As for temperature, the Czech Hydrometeorological Institute (CHMI) provided records of the daily mean temperature during the study period from 14 stations spread across the Czech Republic (Fig. 1). A similar set of stations was used in previous studies analysing the impact of temperature extremes on mortality in Czech Republic (Kyselý and Kříž 2008; Urban et al. 2017). The temperature records were averaged over the stations to account for the daily mean temperature in the whole country.

### Statistical analysis

The statistical analysis was performed using a time series regression. A generalized linear model (GLM) was applied separately on each population group to estimate group-specific temperature-mortality associations. The error was chosen to follow a quasi-Poisson distribution (Wedderburn 1974), particularly adapted to overdispersed count variables such as mortality (Istiana et al. 2020). To take into consideration the delayed and non-linear effects of thermal conditions on mortality, the temperature-mortality association was modelled using a distributed lag non-linear model (DLNM) (Gasparrini et al. 2010). DLNMs consist in introducing in the GLM a cross-basis matrix that combines two functions describing respectively the temperature-mortality and the lag-mortality associations. The temperature-mortality curve was modelled by a quadratic spline with one internal knot placed at the 75th percentile of summer temperature (“percentile of summer temperature” will hereafter be referred to as “percentile”). The lag-mortality curve was controlled by a natural cubic spline with two internal knots equally spaced on the log scale, and the lag period was extended to 14 days. The number and position of knots in the temperature-mortality space as well as the number of lags in the lag-mortality space were chosen after performing a sensitivity analysis (see Supplementary material, Fig. S1). All combinations of number of lags from 7 to 14, and of position of knots (a single knot at the 50th, 75th, and 90th percentiles and two knots at the 50th and 90th percentiles) were tested, and the best model was selected according to the lowest quasi Akaike information criterion (Akaike 1974, Wedderburn 1974, Bolker 2009).

To analyse the short-term associations between temperature and mortality, time series were adjusted for periodic patterns and a long-term trend. The weekly pattern was controlled by the day of the week (DOW), a 7-level factor variable, while the seasonal pattern was controlled by the day of the year (DOY). The DOY was modelled with a natural cubic spline with two degrees of freedom per year. An interaction (denoted as “:” in the formula below) between the spline and the year was also included to allow the degrees of freedom to vary from one year to another. Long-term trend was controlled by a time variable, modelled with a natural cubic spline with two degrees of freedom (one degree per whole decade).

The model design choices were based on models presented in previous papers (Achebak et al. 2018; Ellena et al. 2020a). Using the same algebraic formulation as Ellena et al. (2020a), the final model designed in this study can be described as follows:

$$\log \left(E\left({Y}_t\right)\right)=\alpha + cb+ DO{W}_t+ ns\left( DO{Y}_t, df=2\right): factor\left( yea{r}_t\right)+ ns\left( time, df=1\ per\ decade\right)$$

where *Y*_*t*_ represents the daily count of deaths, *E*(.) is the expected value, *α* is the intercept, *cb* is the cross-basis matrix, and *ns* is a natural spline.

The effect of a given temperature on mortality was reported as a relative risk (RR), which expressed the ratio between the mortality risk at the temperature under analysis over the mortality risk at a reference temperature. The reference temperature was defined as the minimum mortality temperature (MMT), i.e. as a point estimate of the temperature within a restricted range between the 50th and 90th percentiles, for which the overall exposure-response curve reached the minimum value (analogous to Tobías et al. 2017, but we did not calculate the MMTs’ confidence intervals). To characterize the overall effect of temperature over the whole lag period, the overall temperature-mortality association was computed by aggregating all the contributions of each lag. The overall RR at the 99th percentile (hereafter noted “99th RR”) was chosen to represent the risk of mortality among the selected population groups associated with extreme heat (analogically to Gasparrini et al. 2015, Achebak et al. 2018, Marí-Dell’Olmo et al. 2019, Ellena et al. 2020a, Petkova et al. 2021). A significance analysis was performed in order to assess the mortality risks differences between men and women. The statistical significance of the difference between two 99th RRs was evaluated by an interaction test according to Altman and Bland's formula (Altman 2003; Altman and Bland 2011):

$$Z=\frac{E_1-{E}_2}{\sqrt{S{E}_1^2+S{E}_2^2}}$$

where *Z* represents the Z-test, *E*_1_ and *E*_2_ are the log transformations of the 99th RR estimates of the two groups, and *SE*_1_ and *SE*_2_ are their respective standard errors. The difference between two 99th RRs was considered significant for a *p* value smaller than 0.05.

Ultimately, the attributable fraction (AF) and attributable number (AN) of deaths, which respectively represent the portion of deaths among all summer deaths of the study period associated with high temperatures and their absolute number (AN = AF * number of all summer deaths), allowed to express the burden of heat-related mortality upon the total mortality. The AFs and ANs among each population group were calculated for days with daily mean temperature larger than the 95th percentile. Although there is no universally accepted heat wave definition in public health studies, the 95th percentile is generally understood as a threshold of a heat wave according to the WHO and WMO Guidance on Warning System Development (McGregor et al. 2015).

All models and calculations were performed on the software R (version 4.1.2), using the package dlnm (version 2.4.7) developed by Gasparrini (2011) and the function attrdl (attributable risk from distributed lag non-linear models) developed by Gasparrini and Leone (2014).

## Results

The 19 time series representing daily mortality from 1995 to 2019 in the Czech Republic in different population groups were modelled using DLNMs with quasi-Poisson family distribution. For each population group, RRs were obtained for the whole range of summer temperature and for 0 to 14 days of lag. The AF and AN of each group were also computed. Table 1 provides a summary of the number of deaths, the mean age, the MMT, the 99th RR, the AF and the AN of each population group.

**Table 1:** Number of deaths, mean age, minimum mortality temperature (MMT), overall relative risk at the 99th percentile of summer temperature (99th RR), attributable fraction (AF), and attributable number (AN) by population group. The columns “95% CI” specify the 95% confidence intervals of the respective preceding columns

Sex	Category	Total deaths	Mean age	MMT [°C]	99th RR	95% CI	AF [%]	95% CI	AN	95% CI
TOTAL	TOTAL	1,079,302	72.96	19.4	1.20	1.16–1.23	0.70	0.60–0.79	7534	6468–8602
F	TOTAL	529,740	76.90	16.2	1.25	1.21–1.30	0.90	0.77–1.03	4762	4116–5411
M	TOTAL	549,562	69.16	20.0	1.16	1.11–1.20	0.55	0.41–0.68	3002	2281–3715
F	0–64	79,355	51.97	19.9	1.08	0.97–1.20	0.27	-0.07–0.64	215	− 64–499
	65–74	98,722	70.13	15.6	1.09	1.00–1.19	0.36	0.07–0.65	359	84–629
	75–84	181,854	79.87	16.3	1.26	1.18–1.34	0.90	0.69–1.11	1634	1237–2016
	85+	169,809	89.31	15.5	1.42	1.34–1.50	1.50	1.27–1.72	2554	2202–2918
M	0–64	175,866	51.59	18.8	1.11	1.03–1.19	0.38	0.17–0.61	672	278–1061
	65–74	147,135	69.72	20.5	1.11	1.03–1.20	0.39	0.11–0.65	574	204–939
	75–84	150,660	79.33	20.1	1.25	1.16–1.34	0.82	0.55–1.07	1232	832–1612
	85+	75,901	88.57	19.8	1.18	1.07–1.30	0.71	0.34–1.06	540	247–817
F	DIV	54,962	71.01	19.4	1.40	1.25–1.56	1.40	1.01–1.79	767	525–976
	MAR	112,550	67.83	18.4	1.12	1.04–1.22	0.45	0.17–0.74	511	208–807
	SIN	28,222	61.36	15.5	1.20	1.03–1.40	0.69	0.10–1.18	194	51–329
	WID	334,006	82.24	16.0	1.28	1.22–1.34	0.99	0.84–1.16	3314	2809–3848
M	DIV	73,210	62.58	19.5	1.14	1.03–1.26	0.53	0.14–0.88	387	117–650
	MAR	308,241	70.39	20.3	1.14	1.08–1.20	0.46	0.28–0.63	1421	863–1972
	SIN	59,213	50.34	17.8	1.15	1.04–1.28	0.59	0.19–0.94	347	124–573
	WID	108,898	80.32	20.0	1.24	1.14–1.35	0.80	0.47–1.09	867	531–1175

### Sex

**Fig. 2:**
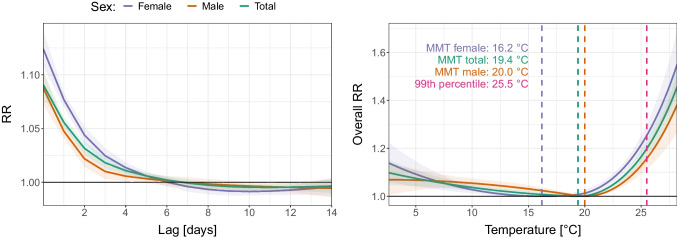
Left: Lag-mortality association between the days of lag and the mortality relative risk (RR) at the 99th percentile of summer temperature (25.5 °C) in the Czech Republic over the period 1995–2019 divided by sex. Right: Overall association between the summer temperature and the mortality relative risk (RR) in the Czech Republic over the period 1995–2019 divided by sex. The purple, orange, and green vertical lines, respectively, represent the minimum mortality temperature (MMT) of women, men, and the total population. The pink vertical line represents the 99th percentile of summer temperature

Figure 2 shows different vulnerability to heat between men and women. Indeed, at the 99th percentile, the risks for women were higher than those for men in the first 7 days after exposure, and the mortality displacement was thus larger afterwards (Fig. 2, left). On the whole lag period, the MMT was found lower by 3.8 °C for women (16.2 °C) than for men (20.0 °C). What is more, women presented a higher overall mortality risk than men at temperatures higher than the MMT (Fig. 2, right).

**Fig. 3:**
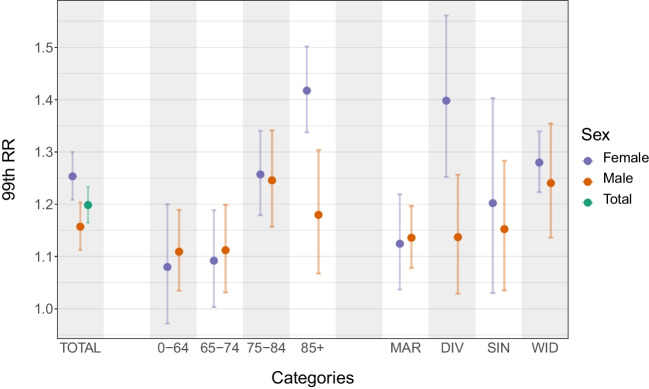
Overall relative risk at the 99th percentile of summer temperature (99th RR) in the Czech Republic over the period 1995–2019 divided by population group. Error bars represent the 95% confidence intervals

**Fig. 4:**
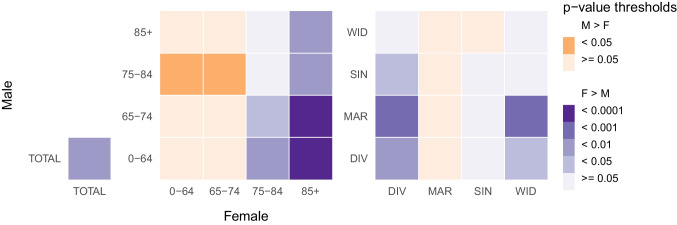
Heatmaps displaying the *p* values of interaction tests determining the statistical significance of the relative risks at the 99th percentile of summer temperature differences between men and women in the total population (left), by age group (middle) and by marital status (right). The *p* values were stratified by thresholds (lower than 1, 0.05, 0.01, 0.001, and 0.0001). The risks difference between two groups was considered significant for *p* values lower than 0.05. Purple (orange) *p* values represent population groups for which women (men) obtained a higher mortality risk than men (women)

At the 99th percentile in particular, the 99th RR was significantly higher (*p* value < 0.01, Figs. 3 and 4) among women (1.25; 95% confidence interval (CI) = 1.21–1.30) than among men (1.16; 95% CI = 1.11–1.20). Sex inequalities in heat vulnerability were also observed through the age and marital status categories. The values of the 99th RRs for women were higher than those for men in five out of eight categories, and those differences were significant for two of them (“85+” and “DIV”). In the three categories for which the 99th RRs were higher for men than for women (“0–64”, “65–74”, and “MAR”), the differences were found insignificant.

### Age

The risks in each age category presented almost no disparity between men and women, except for the “85+” category (Fig. 3). Indeed, for both sexes, the lowest risks were found among people younger than 75 years old (categories “0–64” and “65–74”). In these two age groups, the 99th RRs were slightly higher for men than for women, but the differences were found insignificant (Fig. 4). For women, the risk grew with age, and the highest risk was found among women older than 85 (1.42; 95% CI = 1.34–1.50). In contrast, the highest risk for men was smaller and was found among men between 75 and 84 (1.25; 95% CI = 1.16–1.34). Women above 85 had a much higher risk than men above 85, and the difference between the 99th RRs of these two categories was significant (*p* value < 0.01, Fig. 4): 1.42 (95% CI = 1.34–1.50) for women versus 1.18 (95% CI = 1.07–1.30) for men.

### Marital status

As for marital status, apart from divorced people, the risks in all the other categories were comparable between men and women (Fig. 3). For both sexes, the lowest risk was obtained among married people, but the 99th RR was slightly higher for married men than for married women: 1.14 (95% CI = 1.03–1.26) and 1.12 (95% CI = 1.04–1.22), respectively, although the difference was found insignificant (Fig. 4). Women had the highest risk when they were divorced (1.40; 95% CI = 1.25–1.56), while men had the highest risk when they were widowed (1.24; 95% CI = 1.14–1.35). Divorced women were found especially vulnerable to heat. Indeed, the 99th RR for divorced women was the second largest among all tested population groups, and it was significantly larger (*p* value < 0.01, Fig. 4) than for divorced men: 1.40 (95% CI = 1.25–1.56) versus 1.14 (95% CI = 1.03–1.26).

### Attributable fractions (AF) and attributable numbers (AN)

**Fig. 5:**
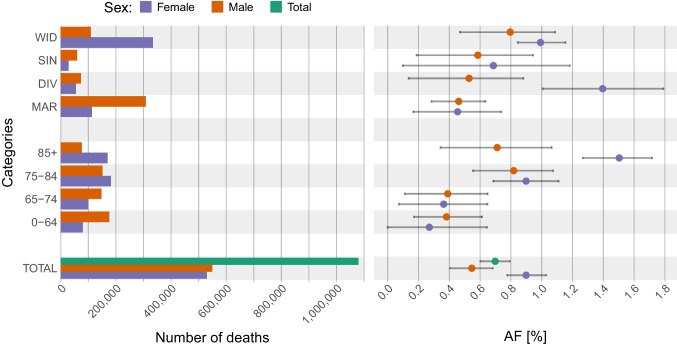
Number of deaths (left) and attributable fraction (AF) (right) by population group in the Czech Republic over the summer months of the period 1995–2019. Error bars represent the 95% confidence intervals

The results of AF (Table 1 and Fig. 5) showed that the deaths which occurred during the summer months of the study period among the total population were attributable to heat in 0.70% (95% CI = 0.60–0.79) of the cases. Women were proportionally more affected by heat than men (0.90%; 95% CI = 0.77–1.03 for women versus 0.55%; 95% CI = 0.41–0.68 for men). The smallest AF was found for women younger than 64 (0.27%; 95% CI = 0–0.64) and the highest for women above 85 (1.50%; 95% CI = 1.27–1.72) and divorced women (1.40%; 95% CI = 1.01–1.79). The AFs were found consistent with the 99th RRs as they ranked the categories in the same order in terms of heat vulnerability.

However, ANs (Table 1) provide a different perspective on the overall burden of heat on the total mortality as they show the actual number of deaths attributable to heat between 1995 and 2019, during the summer months. According to the results, more than 7500 (7534; 95% CI = 6468–8602) deaths that occurred among the whole population during the summer months of the study period were attributable to heat. Among them, more women (4762; 95% CI = 4116–5411) died in association to heat than men (3002; 95% CI = 2281–3715). The specific categories for which a large number of deaths was attributable to heat were the widowed women (3314; 95% CI = 2809–3848) and women above 85 years old (2554; 95% CI = 2202–2918).

## Discussion

The present study assessed the effects of summer temperatures on mortality in the Czech Republic over 25 years, with results categorised by sex, age, and marital status. The 99th RRs obtained for each category ranged between 1.08 and 1.42, which is in line with similar studies using DLNMs in European countries (Gasparrini et al. 2015; Petkova et al. 2021). Studies conducted in Southern Europe usually obtained higher 99th RRs (Achebak et al. 2018; Marí-Dell’Olmo et al. 2019; Ellena et al. 2020a), which is consistent with warmer climate (Urban et al. 2021).

The results obtained in this study confirmed that heat-related mortality risk differed by analysed category. Our findings validate the already found vulnerability for some specific groups, such as for women in respect to men (Fouillet et al. 2006; Stafoggia et al. 2006; Yu et al. 2010; Gabriel and Endlicher 2011; Huang et al. 2015; Achebak et al. 2018; Marí-Dell’Olmo et al. 2019; Son et al. 2019; Ellena et al. 2020a, 2020b), for elder over young people (Canouï-Poitrine et al. 2006; Hajat et al. 2007; Yu et al. 2010; Kenny et al. 2010; Huang et al. 2015; Arbuthnott and Hajat 2017; Watts et al. 2019; Son et al. 2019; Breil et al. 2021; Wan et al. 2022), and for non-married (i.e. single, divorced, widowed) in respect to married people (Stafoggia et al. 2006; Canouï-Poitrine et al. 2006; Fouillet et al. 2006; Gronlund et al. 2015; Ellena et al. 2020a, 2020b; Wan et al. 2022).

What is more, this study brought new results on heat vulnerability at the intersections of sex with other social categories about a Centre European region. Indeed, with regard to age, the highest risk for men was obtained in the 75–84 category, versus the 85+ category for women. By marital status, men were found to be the most at risk when widowed, while women were found at the highest risk when divorced. The risk obtained for divorced women was significantly higher than for divorced men. As far as we know, this is quite a neglected phenomenon, as a similar finding was only reported in one other study (Ellena et al. 2020a). In the sections below, possible causes of the observed differences in heat vulnerability between individual population groups with a focus on gender and marital status differences are discussed.

### Sex and age

Scientific literature suggests that differences in heat vulnerability between sex and age groups might arise from physiological characteristics in thermoregulating abilities between elder men and women. Studies have shown a diminishing ability of thermoregulating the body when growing older (Kenney and Munce 2003) and highlighted the association of advanced age with risk factors such as renal failure, cardiac rhythm disturbance, thrombosis and nervous system dysfunction (McGregor et al. 2015), diabetes, cardiovascular and respiratory diseases (Watts et al. 2019), or the use of medications (Arbuthnott and Hajat 2017). In addition, some studies suggested that women had less capability than men to regulate the temperature of their body under heat stress (Fox et al. 1969; Burse 1979). More recent studies (Charkoudian and Stachenfeld 2014; Yanovich et al. 2020) reported a certain bias and limitations in past research with regard to female thermoregulation and did not find any evidence of a female disadvantage in thermoregulation in situation of exercise or commonly performed activities if factors such as fitness and body size are taken into account. However, as women tend to have in average lower aerobic capacity due to smaller body size, less muscle mass, and more fat mass, they might also be more vulnerable to extreme thermal conditions (Yanovich et al. 2020).

Previous studies found larger vulnerability to heat among people with chronic respiratory and cardiovascular disease (Hajat et al. 2007; Basu 2009; Hanzlíková et al. 2015; Arbuthnott and Hajat 2017), and these findings were confirmed also by the current data (not shown; Vésier 2022). Higher occurrence of chronic disease and poorer health was generally associated with lower socioeconomic levels (Craig 2005; Lawlor and Sterne 2007; Lago et al. 2018; McMaughan et al. 2020). Similarly, although the influence of the socioeconomic level on heat-related mortality was sometimes found insignificant (Urban et al. 2016; Arbuthnott and Hajat 2017; Son et al. 2019), multiple studies (O’Neill et al. 2003; Schwartz 2005; Stafoggia et al. 2006; Basu 2009; Otto et al. 2017; Ellena et al. 2020b; Breil et al. 2021) suggested that low levels of education or low incomes could enhance the vulnerability to heat. Consequently, the larger heat vulnerability of women compared to men may also be partly explained by socioeconomic disparities (Breil et al. 2018).

Such differences might arise from inequalities in the labour market. According to a report by the Czech Statistical Office (CZSO, Czech Statistical Office 2021), the gender pay gap in the Czech Republic was the 5th largest in the European Union in 2018, with men being paid on average 18.9% more than women. This same report also pointed out that women were less professionally active than men, since they were in charge of taking care of children or adults with disabilities in a majority of cases. Women were also more likely to be unemployed (especially when they have children) or to have definite-period or part-time contracts. Consequently, since the pensions’ amount is related to the money earned during active years, female pensioners are disadvantaged: men pensions were about 20% higher than those of women in the Czech Republic in 2010 (Andel 2014). This is in accordance with the fact that among people over 75 years old, 21.6% of women were at risk of poverty or social exclusion in the Czech Republic on average between 2015 and 2019, versus 6.34% of men (Eurostat 2022a).

**Fig. 6:**
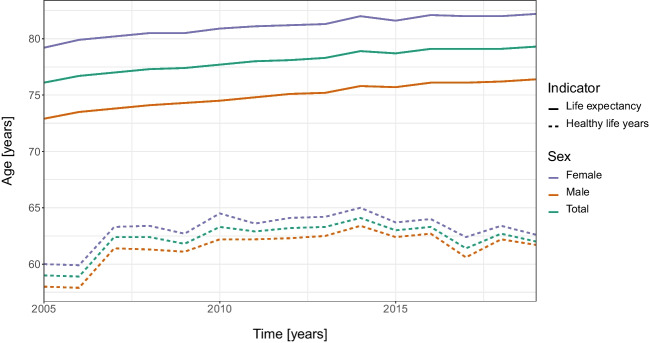
Evolution of the life expectancy and the healthy life years in the Czech Republic over the period 2004–2019 by sex. The data was provided by the European Institute for Gender Equality (EIGE)

Therefore, despite living longer, women might be more vulnerable to heat than men due to generally lower quality of life, higher risk of isolation in the last years of their life (our data show that 96% of women in the category 85+ were non-married at the time of death, compared to 59% of men), and general health gap existing between men and women. Indeed, many studies conducted in Europe showed that, despite having a longer life expectancy, women reported more health conditions than men and had proportionally shorter healthy life years (Oksuzyan et al. 2009; Oksuzyan et al. 2010; Dahlin and Härkönen 2013; Romero-Ortuno et al. 2014; Palència et al. 2017). More research is needed to understand the mechanisms underlying this observation, but several hypotheses including biological or behavioural factors (Oksuzyan et al. 2010), lower socioeconomic positions, or the “double burden” of paid work and (unpaid) household work (Dahlin and Härkönen 2013) were raised. This theory is also supported by data (EIGE 2022) on the evolution of the life expectancy and the healthy life years in the Czech Republic between 2004 and 2009 displayed by sex in Fig. 6. It confirmed that the difference by sex in healthy life years was considerably lower than the one in life expectancy (1.7 and 6.2 years on average, respectively, between 2005 and 2019). Hence, elder women were more likely to live more time in a frail state and therefore to be more susceptible to heat.

### Sex and marital status

The existence of a socioeconomic gap between men and women seems to be especially important when results on heat-related mortality risk according to marital status are considered. In our study, married people were at lower risk of heat-related mortality compared to single, widowed, and divorced. Although widowed people were the oldest among all groups (82 years old, Table 1), single and divorced people were younger than married people (54, 66, and 70 years old, respectively); hence, the largest vulnerability among older population does not fully justify the larger mortality risk of non-married people found in this study.

This was especially true for the group of divorced women. Although they were on average older than divorced men at the time of death (71 and 63 years old, respectively), they were not older than other groups such as widowed women or men (82 and 80 years old, respectively) which yet obtained lower mortality risks. These findings support the hypothesis that differences in heat vulnerability might arise also from other than physiological factors, such as disparities in the respective economic situations of married and non-married people. It was established in numerous studies that married people had lower mortality than non-married people (Hank and Steinbach 2018; Franke and Kulu 2018; Zueras et al. 2020), in that marriage tended to provide a better socioeconomic situation, to encourage healthier behaviours and to prevent from social isolation. Although some studies documented divorced men to be more at risk of health outcomes than divorced women (Hank and Steinbach 2018; Sbarra and Whisman 2022), divorced women were still reporting more health conditions or psychological distress than other women (Haider et al. 2003; Lorenz et al. 2006).

Moreover, as divorced women usually face a larger decrease in their relative income (Symoens et al. 2014; Sbarra and Whisman 2022) and a higher likelihood to obtain the custody of their potential children (Symoens et al. 2014), they are confronted to a larger decline in general living standards than men. In the Czech Republic specifically, 90% of the single adult households with children were run by women in 2013 (CZSO 2015), and on average between 2015 and 2019, 40.8% of the households of one adult with dependent children were at risk of poverty or social exclusion versus 12.28% of the total population (Eurostat 2022b). Consequently, poorer socioeconomic levels, combined with other physiological and demographic factors, may have fostered the larger vulnerability to heat of divorced women.

### Limitations

As far as we know, this is the first study investigating the role of individual socioeconomic level on heat-related mortality in the Czech Republic. Our study provides novel findings on heat-related mortality risks with respect to marital status of the deceased and highlights the potential role of gender inequalities in heat-related mortality. Yet, several limitations need to be acknowledged, mostly related to insufficient data availability.

One limitation of the study lies in the country-level design which does not allow for spatial variations of the temperature-mortality relationships and therefore does not necessarily represent associations existing in different regions. Although information about the place of residence of each deceased was available, previous studies for individual cities and regions other than Prague did not usually provide significant and robust results due to small population samples (Urban et al. 2016), and studies comparing Prague with a rural region did not show many significant differences for heat-related mortality (Urban et al. 2014; Urban and Kyselý 2014). In addition, more precise temperature records were not available at the time of the analysis. Indeed, the gridded temperature dataset used in previous Czech studies (Urban et al. 2016; Urban et al. 2020) was only available until 2017, and the station network used in this study was not dense enough to run a proper spatial analysis. Considering the relatively small area and population of the Czech Republic and the fact that the vast majority lives in the same climate zone (Kottek et al. 2006), using the nation-wide mortality data seems to be a reasonable way to obtain robust and statistically significant results compared with those from other studies.

Another limitation is related to the choice of the categories to analyse, which was delimited by the information provided by the individual mortality data. In addition to the date of death, the age, the sex, and the marital status of the deceased which were used in the present study, the original dataset also stated the education level, which would have been a relevant indicator of the socioeconomic level of the deceased. However, because of significant changes in the encoding and recording of this variable in the death records over the study period, the level of education of a large part of the deceased people was classified as “unknown” after 2013 (see Supplementary material, Fig. S2). For this reason, this category was not consistent enough to be included in the analysis. Other information, such as the income, the professional category, or the number of inhabitants in the household, which would be useful for a better understanding of the impact of the socioeconomic level on heat vulnerability, were not recorded. Therefore, marital status was the best available information about the socioeconomic situation of the deceased.

However, even within the selected variables, one needs to interpret their categorisation with caution, as they only provide a simplified view of reality. In particular, it is worth mentioning that the binary differentiation of sex does not allow to account for all the sex and gender identities existing in the population. Similarly, marital status as a legal denomination does not necessarily represent the actual household situation of the deceased. Our results highlight the critical lack of relevant information in the individual mortality datasets and encourage the relevant authorities for collecting data about educational level and other indicators of socioeconomic status for each deceased. Such information would allow a better insight into the role of individual vulnerabilities and (not only) heat-related mortality risks.

## Conclusions

In this study, the effects of summer (May–September) daily temperature on mortality in the Czech Republic over the period 1995–2019 were modelled using a quasi-Poisson regression model including a distributed lag non-linear model. The study was conducted with a primary focus on sex and gender inequalities; the analysis was consequently disaggregated between men and women, and both groups were further divided by age and, for the first time in a Czech study, marital status.

This study underlined the higher vulnerability to heat of women compared to men, especially among people over 85 years old and, a quite novel finding, among divorced people. These results were discussed to be driven by combinations of physiological, demographical, and socioeconomic factors. The gender-based health and economic inequalities presented in this study highlight the importance of taking into account these factors when collecting data about mortality, identifying vulnerabilities in the society, and planning efficient mitigation and adaptation strategies to prevent the impacts of climate changes on the public health sector.

## Supplementary information

ESssM 1 (DOCX 142 kb)

## Data Availability

Meteorological data used in this study are freely available via the Czech Hydrometeorological Institute's website: https://www.chmi.cz/historicka-data/pocasi/denni-data/Denni-data-dle-z.-123-1998-Sb. The authors are not entitled to share the mortality data with third parties according to the policy of the data provider (the Institute of Health Information and Statistics of the Czech Republic).
