# Supplementary material for: Gender inequalities in heat-related mortality in the Czech Republic
Source: Int J Biometeorol. 2023 Jul 10;67(8):1373–85. doi: 10.1007/s00484-023-02507-2 (PMC10386945; doi:10.1007/s00484-023-02507-2)
Supplement: Supplementary file 1 — (DOCX 142 kb) [file 484_2023_2507_MOESM1_ESM.docx]

**Supplementary material**

Title: Gender inequalities in heat-related mortality in the Czech Republic

Authors: Chloé Vésier, Aleš Urban

**Supplementary figures:**


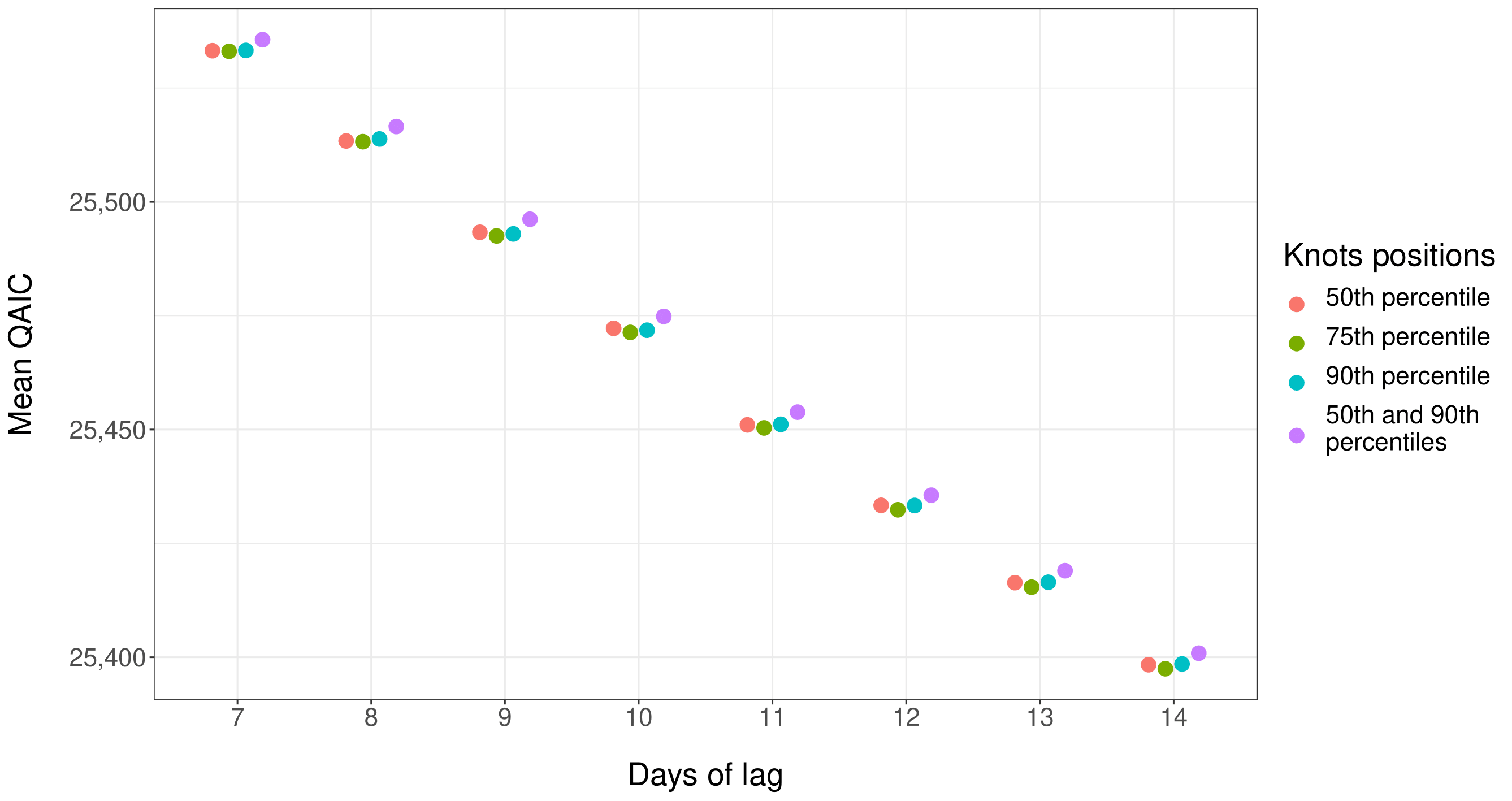


**Fig. S1** Evolution of the quasi-Akaike information criterion (QAIC) averaged over the individual groups by lag and knots position in the temperature-mortality space calculated in the sensitivity analysis. The lowest QAIC was obtained with 14 days of lag and one knot at the 75^th^ percentile of temperature.


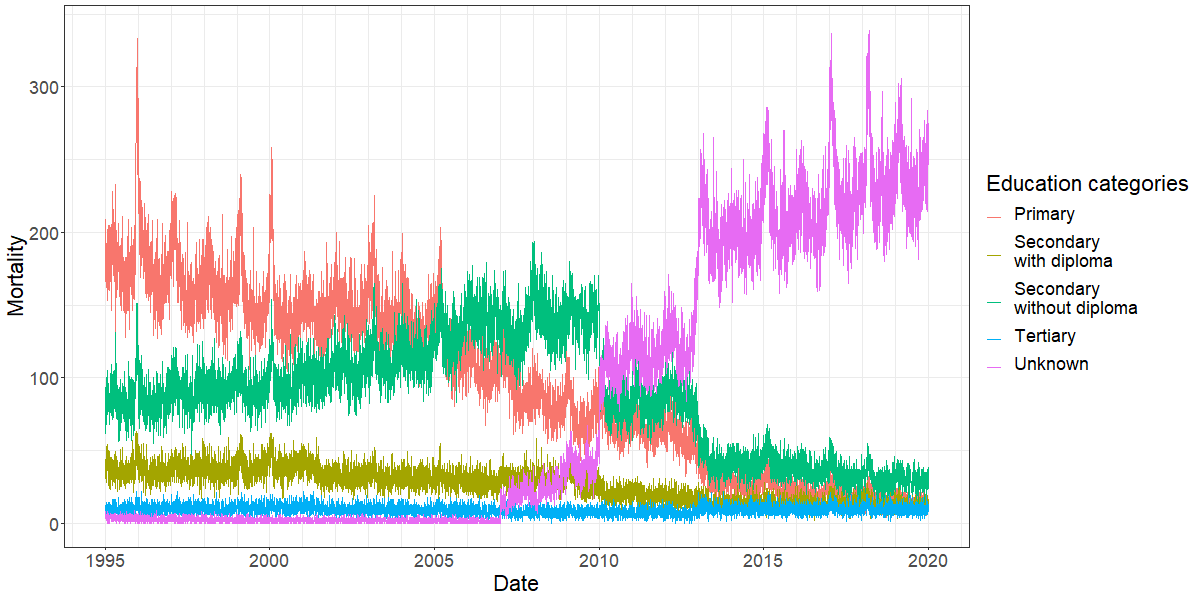


**Fig. S2** Evolution of the daily number of deaths from 1995 to 2019 in the Czech Republic by education category.
